# Protective Factors for Falls Among Independent Older Adults: A Cross-Sectional Study

**DOI:** 10.3390/ijerph22081202

**Published:** 2025-07-31

**Authors:** Warangkana Srimoke, Chamnong Thanapop, Pimpichaya Sangchart, Sopanat Chitpong, Jirasuta Hnoophet, Nattaya Rueangkhanap, Kitipop Jantep

**Affiliations:** 1 Department of Environmental Health and Technology, School of Public Health, Walailak University, Nakhon Si Thammarat 80160, Thailand; sopanat.envi@gmail.com (S.C.); jirasata2544@gmail.com (J.H.); nattaya57482@gmail.com (N.R.); sutima2282@gmail.com (K.J.); 2 Department of Community Public Health, School of Public Health, Walailak University, Nakhon Si Thammarat 80160, Thailand; tchamnon@wu.ac.th; 3 Department of Pharmaceutical Technology, Faculty of Pharmacy, Srinakharinwirot University, Nakhon Nayok 26120, Thailand; pimpichaya@g.swu.ac.th

**Keywords:** protective factors, falls, aged, independent living, cross-sectional studies

## Abstract

As Thailand transitions into a super-aged society, falls are a rising public health issue. However, limited research focuses specifically on independent older adults in rural areas. This study examined intrinsic and extrinsic factors associated with falls among independent older adults in a rural district of southern Thailand, contributing to localized fall prevention strategies. A cross-sectional study was conducted using multi-stage probabilistic sampling with 325 older adults aged 60–79 years residing in Nakhon Si Thammarat. Data were collected through structured interviews, and multivariate logistic regression was used to identify fall predictors. A fall was defined as an unintended fall to a lower level within the previous 12 months. The fall prevalence was 29.8%, with the majority resulting in minor injuries. Multivariate analysis revealed protective factors, including sociodemographic factors such as higher monthly income (adjusted OR = 0.47; 95% CI: 0.30–0.74) and agricultural employment (adjusted OR = 0.50; 95% CI: 0.27–0.95), as well as the extrinsic factor of pet ownership (adjusted OR = 0.53; 95% CI: 0.35–0.81), were significantly associated with reduced fall risk. The study highlights context-specific protective factors that could inform community-based interventions. Future research should assess causality and intervention effectiveness in broader populations.

## 1. Introduction

The global population of individuals aged 60 years and older is expected to nearly double between 2015 and 2050 [1]. Aging is accompanied by a decline in sensory, cognitive, and physical functions, which, alongside chronic diseases common in older adults, increase the risk of falls, a major public health concern [2,3,4]. Globally, falls are the second leading cause of unintentional injury deaths, accounting for an estimated 684,000 deaths each year [5]. While considerable efforts have been made to understand fall risk factors among dependent or frail elderly populations, relatively few studies focus on independent older adults—a group that, although functionally independent, remains vulnerable to fall-related injuries [6,7]. As the risk of falls rises with declining independence in both basic and complex daily activities [8], it is critical to develop a consistent approach for community-based fall prevention programs in this population [9,10]. Intrinsic risk factors such as chronic diseases, medication use, and sensory impairments have been consistently associated with falls [11,12], while extrinsic risks include environmental hazards and lack of home safety measures [13]. However, protective factors—especially context-specific ones—have received less attention, particularly in rural Southeast Asian settings. Previous studies have highlighted the potential role of income, physical labor, and social engagement in fall prevention [14,15,16,17], but their effect among non-dependent older adults in rural Thailand remains underexplored. In this study, we examine these associations within the context of Thailand—a country that has been classified as an aged society since 2005 and is projected to become a super-aged society by 2031 [18,19]. With over 1000 fall-related deaths among older adults annually in Thailand [20], there is an urgent need for effective, targeted fall prevention strategies in aging populations.

Fall risks among older adults differ between urban and rural settings, influenced by a range of contextual and individual-level factors. While research from Nigeria revealed greater falls in urban settings associated with health-related factors such as poor vision and chronic diseases [17], a study conducted in China identified higher fall rates in rural areas due to limited healthcare and poor living conditions [21]. In Northeastern Thailand, sex, family size, congenital disorders, and impaired mobility are significant fall predictors among older adults in home settings [22], whereas among ethnic minority older adults, home environments, fall prevention practices, and mental health conditions significantly impact the risk of falling in northern Thailand [23]. A mixed-methods study in southern Thailand found that female gender, cognitive and sensory impairments, balance issues, semi-dependence, and outdoor toilets were key fall risk factors in older adults. It highlighted the need for targeted prevention, hindered by limited resources, unclear policies, and lack of community awareness [24]. Although falls are recognized as a significant issue affecting older adults, research remains limited, particularly among independent older adults living in rural southern Thailand, an area with distinct social and environmental contexts.

In light of these gaps, the present study aimed to investigate both intrinsic and extrinsic risk factors associated with falls in non-dependent older adults in Nakhon Si Thammarat, southern Thailand. This population represents a distinct subset whose functional capacity and lifestyle may offer insights into contextually appropriate fall prevention strategies. Understanding these factors in context may support the development of targeted, culturally appropriate community interventions to reduce fall risk and promote healthy aging.

## 2. Materials and Methods

### 2.1. Study Design and Setting

This study employed a cross-sectional design and was conducted in Thasala District, a rural coastal area of Nakhon Si Thammarat Province in southern Thailand, between September and December 2023. Nakhon Si Thammarat is the largest province in southern Thailand and has a high population density [25]. Thasala District was purposively selected due to its high proportion of community-dwelling older adults who maintain independence in daily activities.

### 2.2. Population and Sampling

The study population consisted of community-dwelling, non-dependent older adults aged 60–79 years. Eligibility criteria included the ability to perform activities of daily living (ADLs) independently [26], residence in the community for at least 12 months, and the ability to communicate and provide informed consent. Older adults with significant cognitive impairment, debilitating illness, or physical dependence were excluded.

The required sample size was estimated using G*Power version 3.1.9.4 for a logistic regression model with a two-tailed test. The parameters included an odds ratio (OR) of 1.5, a probability of outcome under the null hypothesis Pr(Y = 1|X = 1) of 0.3, a type I error rate (α) of 0.05, and a desired power (1 − β) of 0.95 [27]. Based on these specifications, the minimum required sample size was 328 participants. A total of 325 participants were recruited, yielding a statistical power of approximately 0.947, which was deemed sufficient for the planned analysis. A multistage sampling method was employed:

Stage 1: Thasala District was selected randomly from districts with a large elderly population in the province.

Stage 2: Ten subdistricts within Thasala were selected, and stratified random sampling proportional to the elderly population in each subdistrict was used to select households.

Stage 3: One eligible individual per household was selected using simple random sampling from household rosters provided by local health officers.

### 2.3. Instruments and Measurements

Data were collected using a structured questionnaire developed through an extensive review of existing tools and expert consultation. The questionnaire consisted of three sections.

#### 2.3.1. Sociodemographic, Health-Related Data, and Behavioral Factors

These included sex, age group (60–69 vs. 70–79), marital status, education level, employment type (agricultural vs. non-agricultural, reflecting the rural context [26]), monthly income (≤700 vs. >700 THB) based on the elderly living allowance in Thailand, and living arrangement (live alone vs. living with others). Health-related data included chronic disease count (≤1 type vs. >1 type), including hypertension, dyslipidemia, diabetes, stroke, osteoarthritis, Parkinson’s disease, and dementia; specific health issues (≤1 type vs. >1 type), including knee degeneration, vision impairment, balance disorders, sleep disturbances, walking difficulties, hearing impairment, hunchback posture, cognitive impairment, and hypoesthesia; medication use (yes vs. no) covering the use of antihypertensives, analgesics, hypnotics, diuretics, and sedatives; BMI (normal ≤ 22.9 vs. overweight ≥ 23.0 kg/m^2^); and frequency of nocturia (≤2 times per night vs. >2 times per night). Behavioral factors included frequency of exercise, engagement in social activities, and the ability to perform instrumental activities of daily living (IADL) such as cleaning, cooking, shopping, and others [28].

#### 2.3.2. Fall History

Participants reported the number of falls in the past 12 months, circumstances, time of day, location, and resulting injuries. Falls were defined according to the WHO standard as an event that results in a person coming to rest inadvertently on the ground or floor or other lower level [29]. Responses were categorized into no fall, single fall, and recurrent falls (≥2) [30]. Recall bias in the 12-month fall history was mitigated using structured prompts, though no specific validity study was conducted due to resource constraints [31].

#### 2.3.3. Environmental Assessment

The home environment was assessed via observation and a checklist based on WHO fall-risk environmental factors. Key features included type of flooring (slippery, uneven), presence of obstacles, availability of safety features (e.g., grab bars), toilet type and location, and presence of domestic animals. Interviews were conducted in person by trained research assistants [32,33,34]. The environmental checklist was pilot-tested (n = 30), achieving a Cronbach’s alpha of 0.83 and a Cohen’s kappa of 0.78 [35].

### 2.4. Data Collection Procedure

Data collection was conducted by four trained interviewers with public health backgrounds. The response rate was 92.3% (325/352 eligible participants), with 20 refusals and 7 absent individuals replaced by random selection from the same subdistrict household rosters. Prior to the interview, participants were provided with information sheets, and written informed consent was obtained. Each interview lasted approximately 20–30 min and was conducted in the participant’s home to allow concurrent environmental assessment.

### 2.5. Ethics Approval and Consent to Participate

This study was reviewed and approved by the Human Research Ethics Committee of Walailak University, Thailand (No. WUEC-23-195-01). All study procedures were conducted in accordance with the ethical principles outlined in the Declaration of Helsinki and relevant national regulations. Written informed consent was obtained from all participants after providing detailed information about the study’s objectives, procedures, voluntary participation, and the right to withdraw at any time without consequence.

### 2.6. Data Analysis

All data were analyzed using SPSS version 29.0 (IBM Corp., Armonk, NY, USA). Descriptive statistics (frequencies and percentages) were used to summarize participant characteristics. For inferential analysis, bivariate associations between independent variables and fall incidence were tested using the chi-square test. Variables with *p* < 0.05 were entered into multivariate logistic regression using a backward stepwise method to identify independent predictors of falls. A directed acyclic graph (DAG) was used to select covariates, identifying sociodemographic, health-related, and environmental factors as confounders. Missing data (<5%, primarily income and health issues) were handled using complete-case analysis. Interaction terms assessed effect modification by gender and age group. Adjusted odds ratios (AORs) and 95% confidence intervals (CIs) were reported. Statistical significance was set at *p* < 0.05.

## 3. Results

### 3.1. Characteristic Data

A total of 325 community-dwelling, non-dependent older adults participated in the study. The majority were female (72.3%), aged 60–69 years (52.0%), married (69.2%), and lived with others (92.6%). Most participants had completed primary education (83.4%). Regarding income, 56.3% reported a monthly income of 700 Thai baht or less. Approximately one-quarter (25.2%) were in agricultural work, while 43.3% were employed.

Chronic conditions were common, with hypertension (47.7%) and dyslipidemia (43.7%) being the most prevalent. Over half of the participants were overweight (BMI ≥ 23.0 kg/m^3^) and 37.2% experienced nocturia more than twice per night. A significant proportion (73.2%) did not engage in regular exercise. Despite this, nearly all participants were able to perform both ADLs and IADLs, indicating functional independence. With respect to environmental conditions, 56.0% of households had obstacles in walking paths, 13.5% had inappropriate bed height, and 86.2% lacked grab bars in bathrooms. Approximately 47.1% of participants owned pets, most commonly cats or dogs. Detailed findings are presented in Table S1.

### 3.2. Fall Prevalence and Circumstances

Overall, 97 participants (29.8%) reported experiencing at least one fall in the past 12 months. Among them, 71.1% experienced a single fall, and 28.9% experienced recurrent falls. Falls occurred more frequently among women (82.5%), and recurrent falls were slightly more common in the 70–79 age group. Falls most often occurred around the home (68.0%), particularly in bathrooms (12.4%) and living rooms (12.4%). The most common causes were tripping over obstacles (38.1%) and slipping (30.9%). Most falls resulted in minor injuries, such as bruising (49.5%) or abrasions (32.0%), while only 1% resulted in a severe head injury. Comprehensive findings are provided in Table S2.

### 3.3. Factors Associated with Falls

The intrinsic and extrinsic factors of fall incidence are shown in Table 1. Sociodemographic factors (sex, employment status, and income per month), health-related data (chronic diseases, health issues, and drug use), and environmental factors such as floor obstacles and pets were significantly associated with fall incidence (*p* < 0.05). However, there were no significant associations observed with other sociodemographic factors (age, marital status, education, and living arrangement), health-related data (BMI and nocturia), behavioral factors (exercise, activity engagement, and instrumental activities of daily living), and home environment (location within the house, bed height, reaching for objects, toilet location, toilet type, uneven floors, cleanliness, slippery floors, and grab bars).

### 3.4. Multivariate Logistic Regression

**Figure 1:**
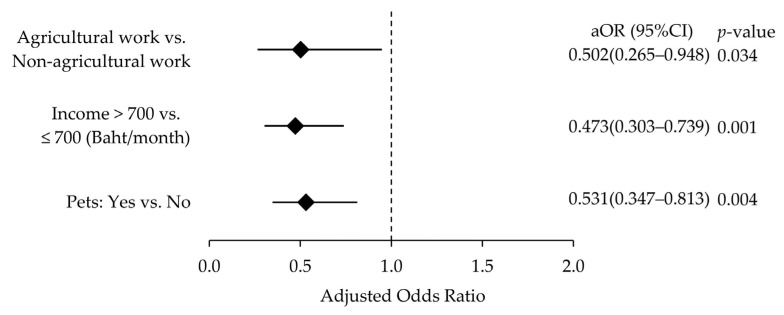
Forest plot showing adjusted odds ratios (aORs) and 95% confidence intervals (CIs) for factors associated with fall risk. Each marker represents the aOR for a given factor, and the horizontal line represents its corresponding 95%CI. aORs less than 1 indicate that all three factors were significantly associated with a reduced risk of falls.

To identify independent predictors of falls, significant variables from the bivariate analysis were included in a multivariate logistic regression model using backward stepwise elimination (Table 2). Participants with income above 700 THB had 52.7% lower odds of falling compared to those with lower income (95% CI: 0.303–0.739, *p* = 0.001). Agricultural workers had a 49.8% reduced risk of falls compared to non-agricultural workers (95% CI: 0.265–0.948, *p* = 0.034). Owning pets was associated with a 46.9% lower likelihood of falling (95% CI: 0.347–0.813, *p* = 0.004). Other variables, including sex, chronic diseases, health issues, medication use, and environmental hazards (e.g., obstacles), did not remain significant in the final model after adjustment. As shown in Figure 1, all three factors were significantly associated with a reduced risk of falls, with adjusted odds ratios below 1 and 95% confidence intervals that did not cross 1.

## 4. Discussion

This study investigated fall risk factors among non-dependent older adults living in a rural area of Southern Thailand. The findings reveal that nearly one-third (29.8%) of participants reported falling in the past year, a rate higher than that reported in many international studies, including those from Republic of Korea (20.9%) [36], Japan (20.8%) [37], and rural China (16.9%) [38]. Few studies have examined falls in rural low- and middle-income country settings outside of Asia. In Latin America, the SABE project reported fall prevalence ranging from 21.6% in Barbados to 34.0% in Santiago, aligning closely with our observed prevalence of 29.8% [39]. Rural studies from Peru and Brazil have also documented high fall rates (25–64%) among older adults, although specific occupational factors were not assessed [40]. A hospital-based study from rural Ghana reported a prevalence of 40.2% and indicated that factors like older age, multimorbidity, and obesity were associated with higher fall risk [41]. Exploration of context-specific factors in diverse rural settings is necessary. These results underscore the urgent need to examine context-specific risk and protective factors in Thailand’s aging population.

### 4.1. Contextualizing the Fall Prevalence

The relatively high fall prevalence in this study may reflect several region-specific factors. Residence in urban or rural settings plays a significant role in fall risk among older adults, with differences reported between regions. In China, falls frequently occur outside of home, with urban residents falling on roads and rural residents in their yards [21]. Conversely, in Northern Greece, the majority of falls occur in indoor areas, especially in bedrooms and bathrooms [42]. In contrast to urban environments, older adults in rural Thailand are more likely to live in homes with physical hazards (e.g., uneven floors, distant toilets, lack of safety installations) and may have less access to fall prevention programs [24]. Nevertheless, participants in this study were independent and functionally independent, suggesting that fall risk persists even in the absence of severe frailty. This aligns with prior research suggesting that falls are not limited to dependent or institutionalized populations but also affect active older adults due to environmental and behavioral factors [38,43]. Therefore, context-specific fall prevention strategies are needed.

### 4.2. Key Protective Factors and Novel Insights

A novel contribution of this study lies in identifying three significant protective factors: higher monthly income, engagement in agricultural work, and pet ownership.

Participants with a monthly income exceeding 700 Thai baht had a significantly lower risk of falling. This finding is consistent with prior studies showing that financial security improves access to healthcare, quality food, and safer living conditions [15,44]. In Thailand, the old-age pension ranges from 600 to 700 baht/month, which is often insufficient for independent living. Therefore, older adults with income above this threshold may enjoy better health maintenance and home safety, thereby lowering fall risk. Previous research in Thailand shows that factors such as poverty and financial status are significant risk factors for falling in older adults and recommends improving home environments to reduce fall risks [23,43]. This finding highlights the socioeconomic dimensions of fall prevention—an area underexplored in fall research.

Older adults engaged in agricultural work were 49.8% less likely to fall compared to those in non-agricultural occupations. Agricultural activity, as a form of informal occupational physical labor, likely promotes muscle strength, joint flexibility, and proprioceptive acuity, serving as a natural and sustained form of exercise. These biological mechanisms enhance postural control, balance, and mobility, thereby reducing the risk of falls [44]. Previous studies have noted that regular physical activity reduces fall risk [45,46], but this study adds a new perspective: informal, occupational physical activity (e.g., farming) may be as protective as structured exercise programs [47]. Consistent with this, prior research in southern Thailand found that employed older adults exhibited a lower risk of falling compared to their unemployed counterparts [24]. This insight has practical relevance for fall prevention in rural and agrarian communities.

Pet ownership, particularly of cats and dogs, was associated with a 46.9% reduced likelihood of falling. This is a relatively underreported finding in the fall literature. Pets may serve as both emotional support—reducing loneliness and depression—and behavioral motivators, encouraging regular movement and engagement with the environment [48,49,50,51]. Interestingly, the Irish longitudinal study on aging found that community-dwelling older individuals who walk their dogs on a regular basis have considerably lower rates of mobility impairment, falls, and fear of falling [52]. Qualitative studies indicate pet owners experience improved mood, sense of safety, and purposeful routine, which may indirectly translate into better balance and physical function [53], while some studies report mixed outcomes depending on pet type and individual circumstances [54]. The present findings suggest that, for independent older adults in rural Thailand, pets may offer protective psychosocial benefits. Further research is needed to explore causality and mechanisms.

### 4.3. Non-Significant Factors and Contrasts with Prior Research

Sociodemographic factors (e.g., sex), health-related data (e.g., chronic diseases, health issues, drug use), and floor obstacles showed no significant associations, even after adjusting for other variables. Past studies consistently show higher fall risk in women, linked to post-menopausal bone loss and frailty, whereas men are more likely to fall due to depression and poor balance [55]. There was no significant association between fall risk and any specific chronic illness. Most independent participants had hypertension or dyslipidemia, with few cases of diabetes. This contrasts with previous studies associating falls with hypertension, diabetes, stroke, osteoarthritis, Parkinson’s, heart disease [33,56], dementia, and depression [4]. Additionally, although previous studies have reported vision impairments as significant risk factors for falls [21], this association was not observed in our findings.

Interestingly, this study did not find significant associations between falls and other commonly cited risk factors such as age, education level, or exercise frequency. This may reflect the relatively high baseline level of independence among the sample, or it may point to the overriding influence of environmental and social supports in this context. The lack of association with age may reflect the relatively narrow age range (60–79 years), as many studies reporting significant age effects include individuals aged 85 and above [4,55]. Still, age-related declines in muscle strength and flexibility remain important contributors to fall risk and mobility impairments [57].

Environmental hazards such as floor obstacles were associated with falls in bivariate analysis but did not remain significant in the adjusted model. This may indicate that the effects of such hazards are mediated or offset by behavioral or socioeconomic factors, such as pet ownership or income, which influence both home environment and fall response. In contrast to previous findings, other extrinsic factors, including lack of cleanliness [45,58], private bedrooms without toilets [43], toilet type [59], reaching for objects in high places [60], and the absence of support features such as grab rails [61], were significantly associated with an increased risk of falls.

### 4.4. Contribution to International Research

This study adds to the global body of knowledge on fall prevention by highlighting protective factors specific to rural, independent older adults in Southeast Asia, demonstrating that functional independence does not eliminate fall risk. Introducing pet ownership and agricultural activity as protective lifestyle components in fall prevention is an angle rarely addressed in prior research. These regional variances not only reflect different living conditions but also highlight the necessity for context-specific fall prevention interventions. Moreover, fall prevention program efforts increasingly depend on collaboration with local and community-based organizations [62]. However, policy gaps may hinder effective implementation, highlighting the need for a clear, evidence-based framework to better align program intentions with practical application and to reduce fall-related risks [63]. As many aging societies face rising rural–urban disparities, findings from this study may inform context-adapted policies that promote safe aging-in-place, not only in Thailand but in other low- and middle-income countries.

### 4.5. Limitations and Strengths

This study has several limitations that should be acknowledged. First, the cross-sectional design limits causal inference; although associations were identified, temporal relationships between risk factors and falls cannot be established. Future longitudinal or interventional studies are needed to confirm causal pathways by tracking incident fall rates over time. Second, fall data were collected through 12-month self-reported recall, which may be subject to memory bias, particularly for minor or non-injurious events. Although a one-month recall period has been suggested for increased accuracy [64], it would not have captured seasonal patterns or recurrent falls. Alternative designs, such as prospective fall diaries or monthly telephone follow-ups, could improve accuracy by capturing real-time data [65]. To mitigate recall bias, interviews were conducted by trained personnel, and participants were encouraged to reflect carefully on recent incidents. Third, while the study employed multistage random sampling and included a reasonably powered sample size, generalizability is limited to independent older adults in rural Thailand. Urban, dependent, or institutionalized populations may face different risk profiles [66]. Finally, while pet ownership was identified as a protective factor, the study did not explore the type, frequency of interaction, or emotional attachment between owners and pets. These contextual nuances should be examined in future mixed-methods or qualitative research.

Despite these limitations, the study has several notable strengths:

- It addresses a research gap in fall risk among functionally independent, rural older adults, a population often underrepresented in fall studies;
- It identifies non-conventional protective factors such as agricultural work and pet ownership that may be more feasible and culturally relevant in rural communities;
- The use of multivariate analysis strengthens the validity of identified associations and provides evidence for targeted policy and intervention development. This study reveals key protective factors that can inform context-sensitive policy development aimed at preventing falls among non-dependent older adults living in rural areas. Importantly, protective elements such as physical activity through agricultural work, financial stability, and emotional support from pet ownership emerged as modifiable factors. These may serve as strategic targets for culturally grounded, community-based fall prevention interventions.

Future research should explore the mechanisms behind these protective factors and evaluate their effectiveness in community-based interventions. By shedding light on the underexplored experiences of non-dependent older adults in rural settings, this study contributes valuable insights for national policy and international aging health discourse, supporting safe and independent aging across diverse populations.

## 5. Conclusions

Falls among independent older adults remain a significant health concern in rural Thailand, with nearly one-third of participants experiencing a fall in the past year. Most falls occurred during daily activities within the home, and though injuries were often minor, the high incidence underscores the need for proactive prevention strategies. Fall risk was significantly associated with intrinsic factors such as sex, employment, monthly income, chronic diseases, health issues, and drug use, as well as extrinsic factors, including obstacles and pet ownership. Importantly, multivariate logistic regression identified three independent protective factors, including higher income, engagement in agricultural work, and pet ownership. These findings suggest that improving financial security, encouraging active daily routines, and promoting emotional and behavioral support through companion animals may help reduce fall risk among community-dwelling older adults. Beyond the Thai context, these findings may have implications for fall prevention strategies in other rural and aging populations worldwide. Effective fall prevention requires targeted interventions and the implementation of evidence-based policies that address clearly identified risk factors. Thailand and many other countries are transitioning into a super-aged society; context-specific fall prevention strategies must be developed. These should integrate economic, environmental, and psychosocial dimensions of aging. The development of community-based fall prevention programs can improve the real-world applicability of interventions and contribute meaningfully to the formulation of effective national policies.

## Figures and Tables

**Table 1:** Participant characteristics and bivariate associations with fall incidence (n = 325).

Variables	Total: n (%)	Falls: n (%)		*p*-Value
		Yes (n = 97)	No (n = 228)	
**Age**				0.915
60–69 years	169 (52.0)	50 (29.6)	119 (70.4)	
70–79 years	156 (48.0)	47 (30.1)	109 (69.9)	
**Sex**				0.008
Male	90 (27.7)	17 (18.9)	73 (81.1)	
Female	235 (72.3)	80 (34.0)	155 (66.0)	
**Employment status**				0.008
Non-agricultural employment	243 (74.8)	82 (33.7)	161 (66.3)	
Agricultural employment	82 (25.2)	15 (18.3)	67 (81.7)	
**Monthly Income**				0.011
≤700 THB	183 (56.3)	65 (35.5)	118 (64.5)	
>700 THB	142 (43.7)	32 (22.5)	110 (77.5)	
**Chronic diseases ^a^**				0.044
≤1	198 (60.9)	51 (25.8)	147 (74.2)	
>1	127 (39.1)	46 (36.2)	81 (63.8)	
**Health issues ^b^**				0.002
≤1	157 (48.3)	34 (21.7)	123 (78.3)	
>1	168 (51.7)	63 (37.5)	105 (62.5)	
**Medication use ^c^**				0.045
No	155 (47.7)	38 (24.5)	117 (75.5)	
Yes	170 (52.3)	59 (34.7)	111 (65.3)	
**Obstacles**				0.018
No	143 (44.0)	33 (23.1)	110 (76.9)	
Yes	182 (56.0)	64 (35.2)	118 (64.8)	
**Pets**				0.035
No	172 (52.9)	60 (34.9)	112 (65.1)	
Yes	153 (47.1)	37 (24.2)	116 (75.8)	

Significant at *p* < 0.05. ^a^ Chronic diseases: hypertension, dyslipidemia, diabetes, stroke, osteoarthritis, and Parkinson’s disease; ^b^ Health issues: knee degeneration, vision impairment, balance disorder, sleep disturbances, walking difficulties, hearing impairment, and hunchback posture; ^c^ Drug use: antihypertensive, analgesic, hypnotic, diuretic, and sedative drugs.

**Table 2:** Multivariate logistic regression analysis of fall risk factors.

Factor	Adjusted Odds Ratio	95% CI	*p*-Value
**Employment status**			
Non-agricultural work	1	-	-
Agricultural work	0.502	0.265–0.948	0.034
**Income per month** (**Thai baht**)			
≤700	1	-	-
>700	0.473	0.303–0.739	0.001
**Pets**			
No	1	-	-
Yes	0.531	0.347–0.813	0.004

## Data Availability

The dataset used and analyzed in the study are available from the corresponding author on reasonable request.

## Supplementary Materials

The following supporting information can be downloaded at: https://www.mdpi.com/article/10.3390/ijerph22081202/s1. Table S1: Sociodemographic characteristics, health-related data, behavioral factors and home environment; Table S2: The characteristics of falls among non-dependent older adults.
