# Supplementary material for: Protective Factors for Falls Among Independent Older Adults: A Cross-Sectional Study
Source: Int J Environ Res Public Health. 2025 Jul 31;22(8):1202. doi: 10.3390/ijerph22081202 (PMC12386102; doi:10.3390/ijerph22081202)
Supplement: Supplementary file 1 [file ijerph-22-01202-s001.zip › ijerph-3728592-supplementary.pdf]

Table S1. Sociodemographic characteristics, Health-related data, behavioral factors and home environment.

Characteristics	n	%
**Sociodemographic characteristics**		
**1. Gender**		
Male	90	27.7
Female	235	72.3
**2. Age (years)**		
60-69	169	52.0
70-79	156	48.0
**3. Marital Status**		
Single/Widowed/Divorced/Separately	100	30.8
Married	225	69.2
**4. Education**		
Illiterate	5	1.5
Primary school	271	83.4
Lower secondary school	9	2.8
Upper secondary school	17	5.2
Diploma/ Vocational Certificate	8	2.5
Bachelor's degree or higher	15	4.6
**5. Employment status**		
Unemployed	141	43.4
Agricultural work	82	25.2
Trading/personal business	79	24.3
General employee	23	7.1
**6. Income per month (Thai bath)**		
≤ 700	183	56.3
> 700	142	43.7
**7. Living arrangement**		
living alone	24	7.4
living with family	180	55.4
living with the spouse	115	35.4
living with relatives	6	1.8
**Health-related data**		
**8. Chronic diseases (> 1 answers)**		
Hypertension	155	47.7
Dyslipidemia	142	43.7
Diabetes	65	20
Stroke	4	1.2
Osteoarthritis	3	0.9
Parkinson's disease	3	0.9
Dementia	0	0.0
**9. Health issue (> 1 answers)**		
Knee degeneration	164	50.5
Vision impairment	152	46.8
Balance disorder	80	24.6
Sleep disturbances	71	21.8
Walking difficulties	69	21.2
Hearing impairment	34	10.5

Table S1. *Cont.*

Characteristics	n	%
Hunchback posture	14	4.3
Cognitive impairment	0	0.0
Hypoesthesia	0	0.0
**10. Drug use (> 1 answers)**		
Antihypertensives	152	46.8
Analgesics	17	5.2
Hypnotics	10	3.1
Diuretics	5	1.5
Sedatives	1	0.3
**11. BMI**		
≤ 22.9 (normal)	149	45.8
≥ 23.0 (overweight)	176	54.2
**12. Nocturia**		
No	51	15.7
Yes (1 time per night)	58	17.8
Yes (≥ 2 times per night)	216	66.5
**Behavioral Factors**		
**13. Exercise (≥30 min)**		
No exercise	186	57.2
Occasional exercise (≤ 2 times/week)	52	16.0
Regular exercise (≥ 3 times/week)	87	26.8
**14. Activity engagement**		
Village health volunteers	11	3.4
Village committee	2	0.6
Elderly Club	10	3.1
Other	15	4.6
No social grouping	287	88.3
**15. Instrumental activities of daily living (IADL) (> 1 answers)**		
Cleaning	257	79.1
Cooking	231	71.1
Shopping	152	46.8
Others	7	2.2
**Home environment**		
**16. Home type**		
Single-story house	236	72.6
Two-story house	63	19.4
House on stilts	26	8.0
**17. Location within the house**		
Upstairs	37	11.4
Downstairs	288	88.6
**18. Location of toilet(near bedroom)**		
No	319	98.2
Yes	6	1.8
**19. Type of toilet**		
Flush toilet	221	68.0
Squat toilet	104	32.0

Table S1. *Cont.*

Characteristics	n	%
**20. Grab bars in the bath room**		
No	280	86.2
Yes	45	13.8
**21. Bed height**		
Non-suitable	44	13.5
Suitable	281	86.5
**22. Items places**		
Items placed too low or too height	21	6.5
Items placed suitable	303	93.5
**23. Uneven floors**		
No	295	90.8
Yes	30	9.2
**24. Cleanliness of floors**		
No	287	88.3
Yes	38	11.7
**25. Slippery floors**		
No	302	92.9
Yes	23	7.1
**26. Obstacle**		
Acceptable	143	44.0
Non-acceptable	182	56.0
**27. Pets Owership**		
No	172	52.9
Yes	153	47.1
**28. Types of pets (> 1 answers)**		
Cat	73	22.5
Dog	70	21.5
Bird	31	9.5
Chicken	30	9.2
Other	14	4.3

Table S2. The characteristics of falls among independent older adults (n=97)

Characteristics	Fall: n(%)	One Fall: n(%)	Recurrent Fall: n(%)
**Sex**			
Male	17 (17.5)	13 (76.5)	4 (23.5)
Female	80 (82.5)	56 (70.0)	24 (30.0)
**Age (years)**			
60–69	50 (51.5)	39 (78.0)	11 (22.0)
70–79	47 (48.5)	30 (63.8)	17 (36.2)
**Location**			
Indoor (bathroom/living room/bedroom/kitchen/stairs)	31 (32.0)	23 (74.2)	8 (25.8)
Outdoor (area around the home)[a]	66 (68.0)	46 (69.7)	20 (30.3)
**Time of day**			
Morning	31 (32.0)	24 (77.4)	7 (22.6)
During the day	63 (64.9)	43 (68.3)	20 (31.7)
Night	3 (3.1)	2 (66.7)	1 (33.3)
**Circumstances**			
Tripping over obstacle	37 (38.1)	22 (59.5)	15 (40.5)
Slip	30 (30.9)	24 (80.0)	6 (20.0)
Misstep	13 (13.4)	9 (69.2)	4 (30.8)
Faint	11 (11.3)	8 (72.7)	3 (27.3)
Falling from a height	6 (6.2)	6 (100.0)	0 (0.0)
**Consequences**			
Injurious (bruises/abrasions/broken bones/head injury)	97 (100.0)	69 (71.1)	28 (28.9)
Non-injurious	0 (0.0)	0 (0.0)	0 (0.0)

[a]: area around the home including the veranda under the eaves and house compound.
